# *catena*-Poly[bis­[octa­kis(dimethyl sulf­oxide)praseodymium(III)] hexa-μ_3_-sulfido-dodeca-μ_2_-sulfido-hexa­sul­fido­hexa­silverhexa­molybdenum]

**DOI:** 10.1107/S1600536808023696

**Published:** 2008-07-31

**Authors:** Zhengjing Jiang, Guodong Tang, Rongqing Li, Yu Zhang

**Affiliations:** a Department of Chemistry, Huaiyin Teachers College, Huai’an 223300, Jiangsu, People’s Republic of China; b Key Laboratory for Soft Chemistry and Functional Materials of the Ministry of Education, Nanjing University of Science and Technology, 200 Xiaolingwei, Nanjing 210094, Jiangsu, People’s Republic of China

## Abstract

The title compound, {[Pr(C_2_H_6_OS)_8_]_2_[Mo_6_Ag_6_S_24_]}_*n*_, contains polymeric Mo_6_S_24_Ag_6_^2−^ anions and [Pr(Me_2_SO)_8_]^3+^ cations, forming a one-dimensional polymeric Mo/S/Ag cluster. The anion assumes the conformation of a zigzag chain. The trivalent cations are arrayed amongst the anionic chains and are well separated from each other. Each Mo and Ag atom is coordinated by four S atoms in a distorted tetra­hedral geometry. The Pr^3+^ atom is coordinated by eight dimethyl sulfoxide ligands, forming a polyhedron-shaped distorted square anti­prism.

## Related literature

The two most relevant known analogs are {[Ca(dmf)_6_][Mo_2_S_8_Ag_2_]}_*n*_ (Yu *et al.*, 1998 ▶) and {[Ca(dmso)_6_]_2_[W_4_S_16_Ag_4_]}_*n*_ (Huang *et al.*, 1996 ▶). For related literature, see: Du *et al.* (1992 ▶); Holloway & Melnik (1993 ▶); Holm (1992 ▶); Hou *et al.* (1996 ▶); Naruta *et al.* (1994 ▶); Niu *et al.* (2004 ▶); Plotnikova *et al.* (2004 ▶); Zhang *et al.* (2001 ▶). 
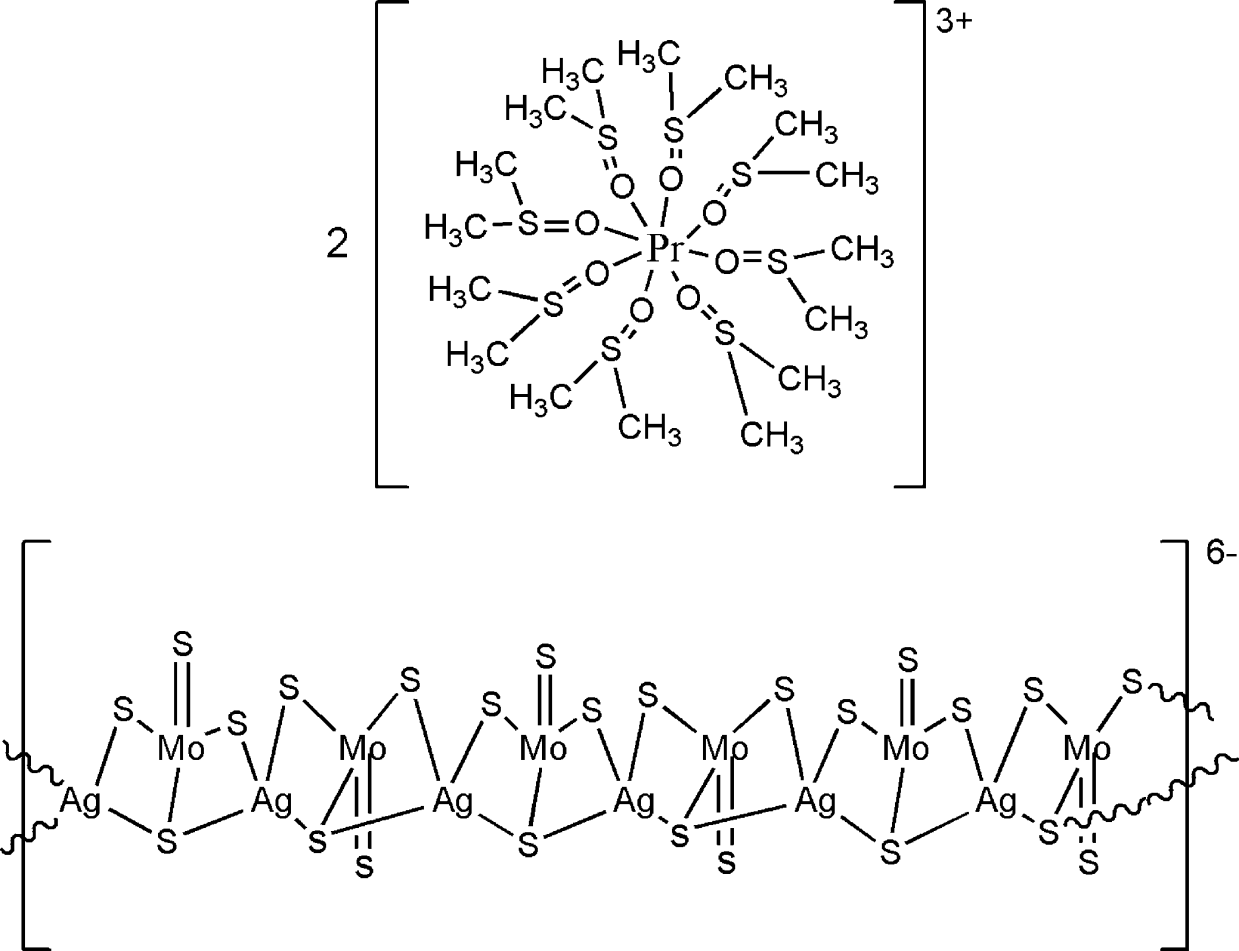


## Experimental

### 

#### Crystal data

[Pr(C_2_H_6_OS)_8_]_2_[Mo_6_Ag_6_S_24_]
*M* *_r_* = 3524.17
Triclinic, 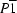

*a* = 15.1808 (15) Å
*b* = 17.6200 (17) Å
*c* = 19.960 (2) Å
α = 89.912 (2)°
β = 89.768 (3)°
γ = 87.616 (3)°
*V* = 5334.4 (9) Å^3^
*Z* = 2
Mo *K*α radiation
μ = 3.47 mm^−1^
*T* = 291 (2) K
0.30 × 0.26 × 0.24 mm

#### Data collection

Bruker SMART APEXII CCD diffractometer
Absorption correction: multi-scan (*SADABS*; Bruker, 2000 ▶) *T*_min_ = 0.375, *T*_max_ = 0.435
63226 measured reflections
19555 independent reflections
16163 reflections with *I* > 2σ(*I*)
*R*_int_ = 0.040

#### Refinement

*R*[*F* ^2^ > 2σ(*F* ^2^)] = 0.049
*wR*(*F* ^2^) = 0.111
*S* = 1.03
19555 reflections
951 parameters
Δρ_max_ = 0.87 e Å^−3^
Δρ_min_ = −1.56 e Å^−3^

### 

Data collection: *APEX2* (Bruker, 2004 ▶); cell refinement: *SAINT* (Bruker, 2004 ▶); data reduction: *SAINT*; program(s) used to solve structure: *SHELXS97* (Sheldrick, 2008 ▶); program(s) used to refine structure: *SHELXL97* (Sheldrick, 2008 ▶); molecular graphics: *SHELXL97*; software used to prepare material for publication: *SHELXL97* and *PLATON* (Spek, 2003 ▶).

## Supplementary Material

Crystal structure: contains datablocks I, global. DOI: 10.1107/S1600536808023696/pv2090sup1.cif

Structure factors: contains datablocks I. DOI: 10.1107/S1600536808023696/pv2090Isup2.hkl

Additional supplementary materials:  crystallographic information; 3D view; checkCIF report

## Figures and Tables

**Fig. 1.**
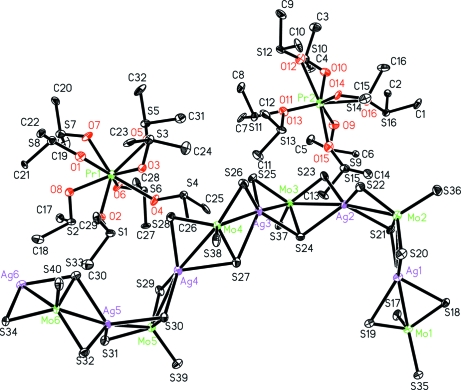
The molecular structure of (I), with atom labels and 30% probability displacement ellipsoids. All H atoms have been omitted.

## supplementary crystallographic information

### Comment

The thiometalates, such as tetrathiotungstate and tetrathiomolybdate, have been extensively used in the synthesis of transition metal sulfides (Holloway *et al.*, 1993), since these moities can surve as multidentate ligands to coordinate with various transithion metals, including Cu, Ag, Au, Zn, Cd, Hg, Fe, Co, Ni, Pd, Pt, Sn and Ru to form a wide range novel structure complexes (Hou *et al.*, 1996; Niu *et al.*, 2004). Up to now, more than 300 heterothiometallic cluster compounds containing these moieties have been synthesized and studied extensively (Zhang *et al.*, 2001). These complexes play a special role in catalysis reactions (Du *et al.*, 1992), biological processes (Holm *et al.*, 1992) and advanced materials (Naruta *et al.*, 1994). Herein, the synthesis and structure of a novel polymeric Mo—Ag—S complex, [Mo_6_S_24_Ag_6_][Pr(dmso)_8_]_2_ (dmso= dimethylsulfoxide) (I), is reported.

The asymmetric unit of (I) comprises polymeric anion (Mo_6_S_24_Ag_6_)_n_^6n-^ and two [Pr(Me_2_SO)_8_]^3+^ cations to form a Mo/S/Ag cluster (Fig. 1). The repeat unit in the anionic chain consists of six butterfly-shaped sub-units joined together by shared silver atoms, which can be viewed as a zigzag chain propagated by cell translation along the *c* -axis. Each Mo atom is coordinated by one terminal S, two *µ*_2_-S and one *µ*_3_-S to form a distorted tetrahedron with S—Mo—S angles varying from 106.70 (7) to 113.42 (6) °. Each Ag atom is coordinated by two*µ*_2_-S, and two*µ*_3_-S to form a distorted tetrahedron, with S—Ag—S angles in the range 89.13 (5) to 142.30 (5) °. The bond distances Mo—S and Ag—S are 2.1156 (18) to 2.2611 (17) Å and 2.4893 (17) to 2.7032 (16) Å, respectively, which are comparable to the values observed in similar compounds (Yu *et al.*, 1998). The Pr^3+^ is coordinated with eight dmso molecules to form a polyhedron shaped like distorted square antiprism, with Pr—O bond lengths in the range 2.379 (5)–2.521 (4) Å, which are similar to the reported values, 2.432 (6) to 2.476 (6) Å, for the related compound (Plotnikova *et al.*, 2004).

### Experimental

AgI (0.235 g, 1 mmol) was added to a solution of (NH_4_)_2_MoS_4_ (0.260 g, 1 mmol in 10 ml dmso) with thorough stir for 20 minutes. The solution underwent an additional stirring for one minute after 0.5 mmol Pr(NO_3_)_3_ . 6H_2_O was added. After filtration the dark-red filtrate was carefully laid on the surface with 10 ml *i*-PrOH. Dark-red crystals were obtained after two weeks.

### Refinement

H atoms were positioned geometrically in a  riding mode with C—H = 0.96 Å and *U*_iso_ = 1.5*U*_eq_ times the methyl C atoms. The highest electron density peak in the final difference map was located close to Pr2 and was deemed meaningless.

### Crystal data

**Table:**

[Pr(C_2_H_6_OS)_8_]_2_[Mo_6_Ag_6_S_24_]	*Z* = 2
*M**_r_* = 3524.17	*F*_000_ = 3416
Triclinic, *P*1	*D*_x_ = 2.194 Mg m^−^^3^
Hall symbol: -P 1	Mo *K*α radiation λ = 0.71073 Å
*a* = 15.1808 (15) Å	Cell parameters from 19555 reflections
*b* = 17.6200 (17) Å	θ = 2.1–25.5º
*c* = 19.960 (2) Å	µ = 3.47 mm^−^^1^
α = 89.912 (2)º	*T* = 291 (2) K
β = 89.768 (3)º	Block, black-red
γ = 87.616 (3)º	0.30 × 0.26 × 0.24 mm
*V* = 5334.4 (9) Å^3^	

### Data collection

**Table:**

Bruker SMART ApexII CCD diffractometer	19555 independent reflections
Radiation source: fine-focus sealed tube	16163 reflections with *I* > 2σ(*I*)
Monochromator: graphite	*R*_int_ = 0.040
*T* = 291(2) K	θ_max_ = 25.5º
phi and ω scans	θ_min_ = 1.2º
Absorption correction: multi-scan(SADABS; Bruker, 2000)	*h* = −18→18
*T*_min_ = 0.375, *T*_max_ = 0.435	*k* = −21→21
63226 measured reflections	*l* = −24→21

### Refinement

**Table:**

Refinement on *F*^2^	Secondary atom site location: difference Fourier map
Least-squares matrix: full with fixed elements per cycle	Hydrogen site location: inferred from neighbouring sites
*R*[*F*^2^ > 2σ(*F*^2^)] = 0.049	H-atom parameters constrained
*wR*(*F*^2^) = 0.111	*w* = 1/[σ^2^(*F*_o_^2^) + (0.070*P*)^2^] where *P* = (*F*_o_^2^ + 2*F*_c_^2^)/3
*S* = 1.03	(Δ/σ)_max_ < 0.001
19555 reflections	Δρ_max_ = 0.87 e Å^−^^3^
951 parameters	Δρ_min_ = −1.56 e Å^−^^3^
Primary atom site location: structure-invariant direct methods	Extinction correction: none

### Special details

**Table:**

Experimental. Yield: 0.377 g in pure form, 63.6% (based on Mo). Analysis calculated for C_32_H_96_Ag_6_Mo_6_O_16_Pr_2_S_40_: C 10.91, H 2.75%; found: C 10.82, H 2.86%.
Geometry. All e.s.d.'s (except the e.s.d. in the dihedral angle between two l.s. planes) are estimated using the full covariance matrix. The cell e.s.d.'s are taken into account individually in the estimation of e.s.d.'s in distances, angles and torsion angles; correlations between e.s.d.'s in cell parameters are only used when they are defined by crystal symmetry. An approximate (isotropic) treatment of cell e.s.d.'s is used for estimating e.s.d.'s involving l.s. planes.
Refinement. Refinement of *F*^2^ against ALL reflections. The weighted *R*-factor *wR* and goodness of fit *S* are based on *F*^2^, conventional *R*-factors *R* are based on *F*, with *F* set to zero for negative *F*^2^. The threshold expression of *F*^2^ > σ(*F*^2^) is used only for calculating *R*-factors(gt) *etc*. and is not relevant to the choice of reflections for refinement. *R*-factors based on *F*^2^ are statistically about twice as large as those based on *F*, and *R*- factors based on ALL data will be even larger.

### Fractional atomic coordinates and isotropic or equivalent isotropic displacement parameters (Å^2^)

**Table:**

	*x*	*y*	*z*	*U*_iso_*/*U*_eq_	
Ag1	0.78546 (3)	0.27180 (3)	0.04430 (3)	0.02200 (10)	
Ag2	0.87061 (3)	0.43334 (3)	0.18316 (2)	0.01931 (10)	
Ag3	0.84246 (3)	0.37447 (3)	0.38218 (2)	0.02125 (10)	
Ag4	0.70699 (3)	0.22873 (3)	0.54426 (3)	0.02098 (10)	
Ag5	0.62965 (3)	0.06681 (3)	0.68251 (2)	0.01838 (10)	
Ag6	0.65666 (3)	0.12879 (3)	0.88251 (3)	0.02199 (10)	
C1	0.8958 (5)	0.9234 (5)	−0.0213 (4)	0.0334 (18)	
H1A	0.9466	0.9536	−0.0169	0.050*	
H1B	0.8691	0.9328	−0.0643	0.050*	
H1C	0.9133	0.8705	−0.0177	0.050*	
C2	0.8145 (4)	1.0521 (4)	0.0348 (4)	0.0246 (14)	
H2A	0.7557	1.0702	0.0239	0.037*	
H2B	0.8540	1.0653	−0.0006	0.037*	
H2C	0.8325	1.0749	0.0758	0.037*	
C3	0.9335 (5)	1.1728 (5)	0.2546 (5)	0.045 (2)	
H3A	0.9868	1.1415	0.2563	0.068*	
H3B	0.9344	1.2104	0.2893	0.068*	
H3C	0.9293	1.1975	0.2117	0.068*	
C4	0.7575 (5)	1.1766 (4)	0.2372 (5)	0.0369 (19)	
H4A	0.7282	1.1535	0.2004	0.055*	
H4B	0.7824	1.2230	0.2225	0.055*	
H4C	0.7158	1.1874	0.2726	0.055*	
C5	0.5570 (6)	1.0542 (4)	0.1992 (4)	0.0377 (19)	
H5A	0.6021	1.0742	0.2270	0.056*	
H5B	0.5083	1.0403	0.2268	0.056*	
H5C	0.5374	1.0922	0.1675	0.056*	
C6	0.5746 (4)	1.0049 (4)	0.0764 (3)	0.0239 (14)	
H6A	0.5765	1.0593	0.0754	0.036*	
H6B	0.5165	0.9901	0.0648	0.036*	
H6C	0.6165	0.9835	0.0448	0.036*	
C7	0.6163 (5)	1.0313 (4)	0.4018 (4)	0.0328 (17)	
H7A	0.6447	1.0789	0.4000	0.049*	
H7B	0.5959	1.0225	0.4467	0.049*	
H7C	0.5670	1.0327	0.3717	0.049*	
C8	0.7746 (5)	0.9889 (4)	0.4284 (4)	0.0312 (16)	
H8A	0.8307	0.9702	0.4115	0.047*	
H8B	0.7666	0.9706	0.4732	0.047*	
H8C	0.7727	1.0434	0.4285	0.047*	
C9	1.0710 (6)	1.0131 (5)	0.3723 (5)	0.044 (2)	
H9A	1.1160	1.0095	0.3383	0.066*	
H9B	1.0976	1.0074	0.4156	0.066*	
H9C	1.0404	1.0618	0.3694	0.066*	
C10	1.0723 (5)	0.8691 (5)	0.3279 (4)	0.039 (2)	
H10A	1.0574	0.8562	0.2827	0.059*	
H10B	1.0725	0.8244	0.3554	0.059*	
H10C	1.1297	0.8901	0.3285	0.059*	
C11	0.7288 (5)	0.7341 (5)	0.3713 (5)	0.042 (2)	
H11A	0.7236	0.6802	0.3756	0.063*	
H11B	0.6798	0.7551	0.3461	0.063*	
H11C	0.7289	0.7568	0.4150	0.063*	
C12	0.9034 (5)	0.7244 (5)	0.3821 (4)	0.0343 (18)	
H12A	0.9443	0.7636	0.3897	0.051*	
H12B	0.9345	0.6798	0.3653	0.051*	
H12C	0.8746	0.7124	0.4235	0.051*	
C13	0.6233 (5)	0.7258 (4)	0.1818 (4)	0.0349 (18)	
H13A	0.6555	0.6992	0.2165	0.052*	
H13B	0.5996	0.6897	0.1514	0.052*	
H13C	0.5760	0.7562	0.2014	0.052*	
C14	0.7488 (5)	0.7190 (4)	0.0897 (4)	0.0303 (16)	
H14A	0.7840	0.7434	0.0566	0.045*	
H14B	0.7065	0.6886	0.0677	0.045*	
H14C	0.7861	0.6872	0.1176	0.045*	
C15	1.0379 (5)	0.7185 (4)	0.1564 (4)	0.0327 (17)	
H15A	1.0847	0.7110	0.1243	0.049*	
H15B	1.0014	0.6753	0.1561	0.049*	
H15C	1.0625	0.7242	0.2002	0.049*	
C16	1.0620 (5)	0.8539 (5)	0.1084 (5)	0.041 (2)	
H16A	1.0395	0.9045	0.0990	0.061*	
H16B	1.0876	0.8321	0.0684	0.061*	
H16C	1.1062	0.8556	0.1426	0.061*	
C17	0.4200 (5)	0.6293 (5)	0.8322 (4)	0.0332 (18)	
H17A	0.4525	0.6748	0.8348	0.050*	
H17B	0.3985	0.6166	0.8760	0.050*	
H17C	0.3711	0.6373	0.8023	0.050*	
C18	0.4629 (6)	0.4856 (5)	0.8743 (5)	0.050 (2)	
H18A	0.5005	0.4405	0.8714	0.075*	
H18B	0.4024	0.4722	0.8713	0.075*	
H18C	0.4724	0.5103	0.9164	0.075*	
C19	0.6012 (6)	0.7740 (5)	0.8559 (5)	0.052 (2)	
H19A	0.6192	0.8171	0.8306	0.078*	
H19B	0.5796	0.7905	0.8990	0.078*	
H19C	0.5553	0.7494	0.8323	0.078*	
C20	0.7616 (5)	0.7735 (5)	0.8928 (4)	0.038 (2)	
H20A	0.8187	0.7482	0.8960	0.058*	
H20B	0.7440	0.7926	0.9361	0.058*	
H20C	0.7642	0.8150	0.8617	0.058*	
C21	0.7209 (4)	0.5185 (4)	0.9328 (3)	0.0260 (15)	
H21A	0.6735	0.5562	0.9299	0.039*	
H21B	0.7415	0.5154	0.9782	0.039*	
H21C	0.7002	0.4701	0.9194	0.039*	
C22	0.8955 (4)	0.4710 (4)	0.9029 (4)	0.0259 (15)	
H22A	0.8879	0.4256	0.8774	0.039*	
H22B	0.8906	0.4597	0.9498	0.039*	
H22C	0.9527	0.4900	0.8937	0.039*	
C23	0.9432 (6)	0.4461 (4)	0.7015 (4)	0.037 (2)	
H23A	0.9331	0.4547	0.7485	0.055*	
H23B	1.0054	0.4408	0.6931	0.055*	
H23C	0.9155	0.4005	0.6882	0.055*	
C24	0.9197 (5)	0.4921 (4)	0.5702 (4)	0.0351 (18)	
H24A	0.9771	0.4671	0.5678	0.053*	
H24B	0.9175	0.5350	0.5405	0.053*	
H24C	0.8759	0.4572	0.5572	0.053*	
C25	0.6809 (4)	0.4519 (4)	0.5293 (4)	0.0243 (15)	
H25A	0.7230	0.4241	0.5565	0.036*	
H25B	0.6940	0.4427	0.4828	0.036*	
H25C	0.6227	0.4356	0.5392	0.036*	
C26	0.6145 (5)	0.5818 (5)	0.4818 (4)	0.041 (2)	
H26A	0.5783	0.6238	0.4983	0.061*	
H26B	0.5776	0.5418	0.4681	0.061*	
H26C	0.6485	0.5982	0.4443	0.061*	
C27	0.4390 (4)	0.6424 (4)	0.6146 (4)	0.0276 (16)	
H27A	0.4616	0.5965	0.5936	0.041*	
H27B	0.3995	0.6691	0.5844	0.041*	
H27C	0.4079	0.6300	0.6549	0.041*	
C28	0.4711 (6)	0.7858 (4)	0.6537 (4)	0.0350 (18)	
H28A	0.4164	0.7749	0.6753	0.053*	
H28B	0.4593	0.8138	0.6132	0.053*	
H28C	0.5057	0.8155	0.6831	0.053*	
C29	0.7501 (5)	0.3295 (5)	0.7443 (4)	0.0303 (16)	
H29A	0.8012	0.3396	0.7178	0.045*	
H29B	0.7423	0.2757	0.7458	0.045*	
H29C	0.7578	0.3482	0.7889	0.045*	
C30	0.5723 (5)	0.3183 (4)	0.7461 (4)	0.0372 (19)	
H30A	0.5997	0.2924	0.7834	0.056*	
H30B	0.5526	0.2817	0.7145	0.056*	
H30C	0.5228	0.3492	0.7617	0.056*	
C31	0.7678 (5)	0.7931 (4)	0.5788 (4)	0.0330 (17)	
H31A	0.8025	0.7561	0.5537	0.050*	
H31B	0.7980	0.8398	0.5805	0.050*	
H31C	0.7117	0.8016	0.5575	0.050*	
C32	0.8554 (5)	0.7855 (5)	0.6919 (4)	0.042 (2)	
H32A	0.8672	0.7631	0.7350	0.063*	
H32B	0.8554	0.8398	0.6955	0.063*	
H32C	0.9002	0.7684	0.6607	0.063*	
Mo1	0.61048 (4)	0.24994 (3)	−0.01700 (3)	0.02195 (12)	
Mo2	0.95462 (4)	0.33692 (3)	0.07937 (3)	0.02121 (12)	
Mo3	0.77241 (4)	0.50147 (3)	0.29754 (3)	0.01952 (11)	
Mo4	0.88146 (3)	0.24889 (3)	0.48144 (3)	0.01828 (11)	
Mo5	0.53974 (4)	0.15946 (3)	0.57924 (3)	0.02027 (12)	
Mo6	0.73016 (3)	0.00388 (3)	0.79719 (3)	0.01753 (11)	
O1	0.7860 (3)	0.5247 (3)	0.8064 (3)	0.0288 (11)	
O2	0.6332 (3)	0.4512 (3)	0.7360 (3)	0.0345 (12)	
O3	0.7990 (3)	0.5091 (3)	0.6592 (2)	0.0267 (10)	
O4	0.6308 (3)	0.5630 (3)	0.6109 (3)	0.0321 (12)	
O5	0.7596 (4)	0.6741 (3)	0.6534 (3)	0.0329 (12)	
O6	0.5575 (3)	0.6695 (3)	0.7031 (2)	0.0272 (11)	
O7	0.7184 (3)	0.6894 (3)	0.7999 (3)	0.0278 (11)	
O8	0.5866 (3)	0.5777 (3)	0.8189 (3)	0.0317 (12)	
O9	0.6996 (3)	0.9815 (3)	0.1620 (3)	0.0352 (12)	
O10	0.8465 (3)	1.0588 (3)	0.2127 (3)	0.0341 (12)	
O11	0.7216 (3)	0.9764 (3)	0.3074 (3)	0.0300 (11)	
O12	0.9460 (3)	0.9716 (3)	0.2996 (3)	0.0313 (11)	
O13	0.8328 (3)	0.8390 (3)	0.3232 (3)	0.0281 (11)	
O14	0.9421 (3)	0.8320 (3)	0.2026 (2)	0.0294 (11)	
O15	0.7539 (3)	0.8055 (3)	0.1910 (3)	0.0347 (12)	
O16	0.8656 (3)	0.9315 (3)	0.1070 (3)	0.0330 (12)	
Pr1	0.68424 (3)	0.57914 (2)	0.72542 (2)	0.02548 (8)	
Pr2	0.82416 (3)	0.92341 (2)	0.22508 (2)	0.02798 (9)	
S1	0.80876 (11)	0.54420 (9)	0.87912 (9)	0.0248 (3)	
S2	0.65104 (11)	0.37779 (10)	0.70611 (9)	0.0258 (4)	
S3	0.89809 (11)	0.52398 (10)	0.65518 (9)	0.0241 (3)	
S4	0.68607 (10)	0.54836 (9)	0.54593 (9)	0.0216 (3)	
S5	0.75131 (11)	0.75815 (9)	0.66337 (10)	0.0273 (4)	
S6	0.52875 (10)	0.70130 (9)	0.63470 (8)	0.0204 (3)	
S7	0.68841 (11)	0.71208 (10)	0.86610 (10)	0.0268 (4)	
S8	0.49025 (11)	0.55351 (10)	0.80179 (9)	0.0283 (4)	
S9	0.60037 (11)	0.97252 (9)	0.15556 (8)	0.0222 (3)	
S10	0.84069 (12)	1.11499 (10)	0.26649 (10)	0.0304 (4)	
S11	0.69190 (12)	0.95725 (9)	0.37809 (9)	0.0253 (3)	
S12	0.99262 (11)	0.93770 (10)	0.35972 (9)	0.0270 (4)	
S13	0.83075 (11)	0.75346 (10)	0.32827 (10)	0.0280 (4)	
S14	0.97585 (10)	0.79828 (9)	0.13613 (8)	0.0218 (3)	
S15	0.69540 (11)	0.78562 (11)	0.13722 (10)	0.0298 (4)	
S16	0.81670 (11)	0.94834 (9)	0.04462 (8)	0.0222 (3)	
S17	0.54376 (12)	0.35754 (9)	−0.01073 (9)	0.0262 (4)	
S18	0.73261 (11)	0.25822 (9)	−0.08048 (9)	0.0228 (3)	
S19	0.64721 (10)	0.21231 (9)	0.08602 (9)	0.0221 (3)	
S20	0.93337 (11)	0.21408 (9)	0.07184 (10)	0.0273 (4)	
S21	0.82979 (10)	0.40873 (9)	0.06364 (8)	0.0212 (3)	
S22	1.01469 (11)	0.35874 (10)	0.17709 (9)	0.0255 (4)	
S23	0.84212 (12)	0.56812 (9)	0.22337 (9)	0.0276 (4)	
S24	0.76903 (11)	0.37773 (9)	0.27082 (9)	0.0229 (3)	
S25	0.82802 (11)	0.51972 (10)	0.39758 (9)	0.0255 (4)	
S26	0.97938 (11)	0.32594 (10)	0.43910 (9)	0.0257 (4)	
S27	0.76187 (11)	0.24381 (9)	0.41848 (9)	0.0228 (3)	
S28	0.84592 (11)	0.28735 (9)	0.58351 (9)	0.0230 (3)	
S29	0.55611 (11)	0.28247 (9)	0.57021 (9)	0.0234 (3)	
S30	0.66753 (10)	0.09053 (9)	0.56254 (8)	0.0211 (3)	
S31	0.48045 (10)	0.13744 (9)	0.67810 (9)	0.0227 (3)	
S32	0.66278 (12)	−0.06600 (9)	0.72356 (9)	0.0265 (4)	
S33	0.72800 (10)	0.12826 (9)	0.76892 (9)	0.0220 (3)	
S34	0.67491 (10)	−0.01717 (9)	0.89779 (8)	0.0178 (3)	
S35	0.51836 (10)	0.16917 (9)	−0.05732 (9)	0.0220 (3)	
S36	1.04706 (12)	0.36546 (11)	0.00374 (10)	0.0309 (4)	
S37	0.63694 (10)	0.54508 (10)	0.29967 (9)	0.0253 (4)	
S38	0.94174 (12)	0.13672 (10)	0.48825 (9)	0.0280 (4)	
S39	0.44672 (12)	0.12648 (11)	0.50451 (10)	0.0304 (4)	
S40	0.86602 (10)	−0.03442 (10)	0.79993 (9)	0.0250 (4)	

### Atomic displacement parameters (Å^2^)

**Table:**

	*U*^11^	*U*^22^	*U*^33^	*U*^12^	*U*^13^	*U*^23^
Ag1	0.0213 (2)	0.0185 (2)	0.0261 (3)	0.00145 (18)	−0.0051 (2)	−0.00202 (19)
Ag2	0.0233 (2)	0.0186 (2)	0.0162 (2)	−0.00268 (18)	0.00113 (18)	−0.00311 (18)
Ag3	0.0201 (2)	0.0245 (2)	0.0188 (2)	0.00338 (18)	−0.00084 (19)	0.00360 (19)
Ag4	0.0194 (2)	0.0228 (2)	0.0205 (3)	0.00222 (18)	0.00016 (19)	0.00386 (19)
Ag5	0.0162 (2)	0.0221 (2)	0.0168 (2)	−0.00022 (17)	−0.00191 (18)	0.00371 (19)
Ag6	0.0211 (2)	0.0185 (2)	0.0262 (3)	0.00178 (18)	−0.0051 (2)	−0.0019 (2)
C1	0.022 (3)	0.055 (5)	0.022 (4)	0.015 (3)	−0.010 (3)	−0.019 (3)
C2	0.023 (3)	0.024 (3)	0.027 (4)	−0.002 (3)	0.009 (3)	−0.010 (3)
C3	0.028 (4)	0.053 (5)	0.055 (6)	−0.001 (4)	−0.011 (4)	0.005 (4)
C4	0.031 (4)	0.024 (4)	0.056 (5)	0.000 (3)	−0.021 (4)	−0.005 (3)
C5	0.049 (5)	0.030 (4)	0.033 (4)	0.003 (3)	0.007 (4)	−0.021 (3)
C6	0.024 (3)	0.026 (3)	0.022 (4)	−0.008 (3)	0.004 (3)	−0.003 (3)
C7	0.037 (4)	0.035 (4)	0.026 (4)	0.008 (3)	−0.015 (3)	−0.018 (3)
C8	0.034 (4)	0.035 (4)	0.024 (4)	0.012 (3)	0.001 (3)	−0.014 (3)
C9	0.042 (5)	0.047 (5)	0.044 (5)	−0.019 (4)	0.007 (4)	0.015 (4)
C10	0.033 (4)	0.049 (5)	0.034 (5)	0.017 (4)	0.019 (3)	0.017 (4)
C11	0.037 (4)	0.042 (5)	0.048 (5)	−0.010 (4)	0.014 (4)	0.003 (4)
C12	0.021 (3)	0.045 (4)	0.038 (4)	−0.009 (3)	−0.001 (3)	0.019 (4)
C13	0.024 (3)	0.026 (4)	0.054 (5)	−0.005 (3)	0.023 (3)	−0.005 (3)
C14	0.035 (4)	0.019 (3)	0.037 (4)	0.001 (3)	0.009 (3)	−0.002 (3)
C15	0.030 (4)	0.022 (3)	0.046 (5)	0.007 (3)	−0.010 (3)	−0.006 (3)
C16	0.030 (4)	0.042 (5)	0.053 (5)	−0.016 (3)	−0.014 (4)	0.006 (4)
C17	0.025 (4)	0.049 (5)	0.026 (4)	−0.008 (3)	−0.012 (3)	0.017 (3)
C18	0.051 (5)	0.048 (5)	0.054 (6)	−0.027 (4)	−0.002 (4)	0.004 (4)
C19	0.038 (5)	0.054 (5)	0.060 (6)	0.033 (4)	0.012 (4)	0.015 (5)
C20	0.046 (5)	0.045 (5)	0.026 (4)	−0.025 (4)	0.019 (3)	−0.014 (3)
C21	0.028 (3)	0.033 (4)	0.017 (3)	−0.013 (3)	0.001 (3)	0.011 (3)
C22	0.019 (3)	0.037 (4)	0.021 (4)	−0.001 (3)	−0.008 (3)	−0.001 (3)
C23	0.052 (5)	0.020 (3)	0.039 (5)	−0.016 (3)	−0.030 (4)	0.008 (3)
C24	0.038 (4)	0.033 (4)	0.032 (4)	0.018 (3)	0.007 (3)	−0.011 (3)
C25	0.021 (3)	0.028 (3)	0.026 (4)	−0.019 (3)	0.006 (3)	−0.006 (3)
C26	0.032 (4)	0.057 (5)	0.034 (5)	−0.016 (4)	0.011 (3)	0.019 (4)
C27	0.019 (3)	0.032 (4)	0.032 (4)	−0.012 (3)	−0.011 (3)	0.000 (3)
C28	0.063 (5)	0.012 (3)	0.029 (4)	0.012 (3)	0.017 (4)	0.003 (3)
C29	0.026 (3)	0.042 (4)	0.022 (4)	0.007 (3)	0.001 (3)	0.013 (3)
C30	0.033 (4)	0.033 (4)	0.046 (5)	−0.002 (3)	−0.020 (4)	0.016 (4)
C31	0.041 (4)	0.016 (3)	0.043 (5)	0.001 (3)	−0.005 (3)	−0.001 (3)
C32	0.026 (4)	0.061 (5)	0.039 (5)	0.016 (4)	−0.021 (3)	−0.024 (4)
Mo1	0.0212 (3)	0.0183 (3)	0.0262 (3)	0.0016 (2)	−0.0051 (2)	−0.0019 (2)
Mo2	0.0216 (3)	0.0202 (3)	0.0215 (3)	0.0035 (2)	−0.0024 (2)	0.0008 (2)
Mo3	0.0198 (3)	0.0192 (3)	0.0195 (3)	−0.0004 (2)	−0.0046 (2)	0.0017 (2)
Mo4	0.0179 (3)	0.0178 (3)	0.0190 (3)	0.0024 (2)	0.0007 (2)	0.0024 (2)
Mo5	0.0198 (3)	0.0196 (3)	0.0210 (3)	0.0037 (2)	−0.0003 (2)	0.0017 (2)
Mo6	0.0201 (3)	0.0186 (3)	0.0136 (3)	0.0026 (2)	0.0000 (2)	0.0006 (2)
O1	0.034 (3)	0.016 (2)	0.035 (3)	0.0028 (19)	−0.002 (2)	−0.001 (2)
O2	0.030 (3)	0.036 (3)	0.038 (3)	−0.009 (2)	−0.007 (2)	0.003 (2)
O3	0.026 (2)	0.032 (3)	0.022 (3)	−0.004 (2)	−0.001 (2)	−0.004 (2)
O4	0.031 (3)	0.044 (3)	0.020 (3)	0.006 (2)	−0.002 (2)	−0.015 (2)
O5	0.044 (3)	0.022 (2)	0.033 (3)	0.001 (2)	0.004 (2)	0.005 (2)
O6	0.027 (2)	0.028 (2)	0.026 (3)	0.007 (2)	−0.017 (2)	−0.009 (2)
O7	0.025 (2)	0.025 (2)	0.033 (3)	0.0097 (19)	−0.018 (2)	−0.009 (2)
O8	0.025 (2)	0.044 (3)	0.027 (3)	−0.006 (2)	−0.006 (2)	0.004 (2)
O9	0.035 (3)	0.032 (3)	0.039 (3)	−0.002 (2)	−0.011 (2)	0.008 (2)
O10	0.022 (2)	0.032 (3)	0.047 (4)	0.000 (2)	−0.001 (2)	0.004 (2)
O11	0.036 (3)	0.026 (3)	0.028 (3)	−0.004 (2)	0.003 (2)	0.006 (2)
O12	0.034 (3)	0.028 (3)	0.031 (3)	0.010 (2)	−0.006 (2)	−0.004 (2)
O13	0.033 (3)	0.019 (2)	0.032 (3)	0.0003 (19)	0.003 (2)	0.008 (2)
O14	0.028 (3)	0.043 (3)	0.017 (3)	0.000 (2)	−0.004 (2)	−0.013 (2)
O15	0.033 (3)	0.035 (3)	0.035 (3)	0.009 (2)	0.005 (2)	−0.009 (2)
O16	0.032 (3)	0.039 (3)	0.027 (3)	0.008 (2)	−0.008 (2)	0.004 (2)
Pr1	0.02798 (19)	0.02676 (19)	0.0212 (2)	0.00459 (15)	−0.00136 (15)	0.00115 (15)
Pr2	0.0321 (2)	0.02562 (19)	0.0257 (2)	0.00574 (16)	−0.00071 (16)	−0.00028 (16)
S1	0.0262 (8)	0.0230 (8)	0.0251 (9)	−0.0015 (6)	−0.0049 (7)	0.0015 (7)
S2	0.0244 (8)	0.0251 (8)	0.0284 (10)	−0.0082 (7)	−0.0040 (7)	0.0006 (7)
S3	0.0207 (8)	0.0279 (8)	0.0233 (9)	0.0053 (6)	−0.0046 (6)	−0.0015 (7)
S4	0.0202 (7)	0.0227 (8)	0.0218 (8)	0.0017 (6)	−0.0002 (6)	−0.0022 (6)
S5	0.0287 (9)	0.0208 (8)	0.0327 (10)	−0.0032 (7)	−0.0028 (7)	−0.0023 (7)
S6	0.0219 (8)	0.0212 (7)	0.0180 (8)	0.0010 (6)	−0.0008 (6)	0.0028 (6)
S7	0.0173 (8)	0.0297 (9)	0.0334 (10)	0.0008 (6)	−0.0076 (7)	0.0049 (7)
S8	0.0236 (8)	0.0350 (9)	0.0273 (10)	−0.0140 (7)	−0.0017 (7)	−0.0016 (7)
S9	0.0266 (8)	0.0192 (7)	0.0203 (8)	0.0062 (6)	−0.0021 (6)	0.0006 (6)
S10	0.0344 (9)	0.0290 (9)	0.0280 (10)	−0.0033 (7)	−0.0056 (8)	0.0063 (7)
S11	0.0310 (9)	0.0228 (8)	0.0217 (9)	0.0033 (7)	0.0042 (7)	−0.0074 (7)
S12	0.0174 (8)	0.0342 (9)	0.0297 (10)	−0.0041 (7)	0.0018 (7)	0.0030 (7)
S13	0.0213 (8)	0.0292 (9)	0.0339 (10)	−0.0074 (7)	−0.0033 (7)	0.0076 (8)
S14	0.0226 (8)	0.0272 (8)	0.0153 (8)	0.0022 (6)	0.0037 (6)	0.0017 (6)
S15	0.0209 (8)	0.0320 (9)	0.0366 (11)	−0.0013 (7)	−0.0033 (7)	0.0024 (8)
S16	0.0253 (8)	0.0223 (8)	0.0193 (8)	−0.0040 (6)	−0.0031 (6)	0.0006 (6)
S17	0.0351 (9)	0.0212 (8)	0.0212 (9)	0.0100 (7)	−0.0031 (7)	−0.0023 (7)
S18	0.0228 (8)	0.0234 (8)	0.0219 (9)	0.0035 (6)	−0.0010 (6)	−0.0073 (7)
S19	0.0213 (8)	0.0186 (7)	0.0263 (9)	0.0015 (6)	−0.0050 (6)	−0.0018 (6)
S20	0.0270 (8)	0.0195 (8)	0.0344 (10)	0.0094 (6)	−0.0046 (7)	−0.0027 (7)
S21	0.0223 (8)	0.0189 (7)	0.0220 (9)	0.0054 (6)	−0.0054 (6)	−0.0059 (6)
S22	0.0211 (8)	0.0324 (9)	0.0230 (9)	−0.0001 (7)	−0.0045 (7)	0.0024 (7)
S23	0.0349 (9)	0.0207 (8)	0.0272 (9)	−0.0025 (7)	0.0124 (7)	−0.0014 (7)
S24	0.0251 (8)	0.0219 (8)	0.0221 (9)	−0.0071 (6)	−0.0052 (7)	0.0035 (6)
S25	0.0266 (8)	0.0284 (8)	0.0216 (9)	0.0002 (7)	−0.0084 (7)	−0.0047 (7)
S26	0.0179 (7)	0.0302 (9)	0.0291 (10)	−0.0012 (6)	−0.0018 (7)	0.0068 (7)
S27	0.0206 (7)	0.0274 (8)	0.0200 (8)	0.0033 (6)	−0.0009 (6)	0.0006 (7)
S28	0.0274 (8)	0.0203 (8)	0.0211 (9)	0.0011 (6)	−0.0011 (7)	−0.0036 (6)
S29	0.0236 (8)	0.0195 (8)	0.0262 (9)	0.0071 (6)	0.0034 (7)	0.0077 (7)
S30	0.0213 (7)	0.0225 (8)	0.0192 (8)	0.0022 (6)	0.0027 (6)	−0.0013 (6)
S31	0.0210 (8)	0.0233 (8)	0.0232 (9)	0.0071 (6)	0.0015 (6)	−0.0030 (7)
S32	0.0344 (9)	0.0199 (8)	0.0255 (9)	−0.0028 (7)	−0.0063 (7)	−0.0032 (7)
S33	0.0214 (8)	0.0184 (7)	0.0262 (9)	0.0016 (6)	−0.0050 (7)	−0.0019 (6)
S34	0.0201 (7)	0.0189 (7)	0.0142 (8)	0.0026 (6)	0.0001 (6)	0.0006 (6)
S35	0.0214 (8)	0.0188 (7)	0.0257 (9)	0.0019 (6)	−0.0050 (6)	−0.0020 (7)
S36	0.0289 (9)	0.0324 (9)	0.0312 (10)	0.0002 (7)	0.0071 (8)	−0.0019 (8)
S37	0.0173 (7)	0.0369 (9)	0.0215 (9)	0.0016 (7)	−0.0041 (6)	−0.0051 (7)
S38	0.0360 (9)	0.0276 (9)	0.0190 (9)	0.0155 (7)	0.0066 (7)	−0.0004 (7)
S39	0.0285 (9)	0.0338 (9)	0.0291 (10)	−0.0009 (7)	−0.0083 (7)	−0.0068 (8)
S40	0.0185 (8)	0.0364 (9)	0.0190 (9)	0.0101 (7)	0.0043 (6)	0.0034 (7)

### Geometric parameters (Å, °)

**Table:**

Ag1—S20	2.4893 (17)	C20—S7	1.673 (7)
Ag1—S19	2.5226 (16)	C20—H20A	0.9600
Ag1—S21	2.5619 (16)	C20—H20B	0.9600
Ag1—S18	2.6341 (17)	C20—H20C	0.9600
Ag1—Mo2	2.9433 (6)	C21—S1	1.781 (6)
Ag1—Mo1	2.9690 (6)	C21—H21A	0.9600
Ag2—S22	2.5072 (17)	C21—H21B	0.9600
Ag2—S21	2.5101 (17)	C21—H21C	0.9600
Ag2—S23	2.5274 (17)	C22—S1	1.867 (7)
Ag2—S24	2.5492 (16)	C22—H22A	0.9600
Ag2—Mo2	2.9349 (6)	C22—H22B	0.9600
Ag2—Mo3	2.9509 (6)	C22—H22C	0.9600
Ag3—S24	2.4901 (17)	C23—S3	1.769 (7)
Ag3—S26	2.4921 (17)	C23—H23A	0.9600
Ag3—S25	2.5783 (18)	C23—H23B	0.9600
Ag3—S27	2.7465 (17)	C23—H23C	0.9600
Ag3—Mo3	2.9660 (6)	C24—S3	1.813 (7)
Ag3—Mo4	3.0099 (6)	C24—H24A	0.9600
Ag4—S29	2.4946 (16)	C24—H24B	0.9600
Ag4—S28	2.5151 (16)	C24—H24C	0.9600
Ag4—S30	2.5571 (16)	C25—S4	1.737 (6)
Ag4—S27	2.6584 (17)	C25—H25A	0.9600
Ag4—Mo5	2.9436 (6)	C25—H25B	0.9600
Ag4—Mo4	2.9602 (6)	C25—H25C	0.9600
Ag5—S30	2.4992 (17)	C26—S4	1.767 (8)
Ag5—S32	2.5100 (17)	C26—H26A	0.9600
Ag5—S31	2.5404 (16)	C26—H26B	0.9600
Ag5—S33	2.5568 (16)	C26—H26C	0.9600
Ag5—Mo5	2.9333 (6)	C27—S6	1.794 (6)
Ag5—Mo6	2.9457 (6)	C27—H27A	0.9600
Ag6—S35^i^	2.4929 (17)	C27—H27B	0.9600
Ag6—S33	2.5085 (18)	C27—H27C	0.9600
Ag6—S34	2.5929 (15)	C28—S6	1.737 (7)
Ag6—S18^i^	2.7032 (16)	C28—H28A	0.9600
Ag6—Mo6	2.9632 (6)	C28—H28B	0.9600
Ag6—Mo1^i^	2.9915 (6)	C28—H28C	0.9600
C1—S16	1.820 (7)	C29—S2	1.862 (7)
C1—H1A	0.9600	C29—H29A	0.9600
C1—H1B	0.9600	C29—H29B	0.9600
C1—H1C	0.9600	C29—H29C	0.9600
C2—S16	1.837 (7)	C30—S2	1.805 (8)
C2—H2A	0.9600	C30—H30A	0.9600
C2—H2B	0.9600	C30—H30B	0.9600
C2—H2C	0.9600	C30—H30C	0.9600
C3—S10	1.788 (8)	C31—S5	1.815 (8)
C3—H3A	0.9600	C31—H31A	0.9600
C3—H3B	0.9600	C31—H31B	0.9600
C3—H3C	0.9600	C31—H31C	0.9600
C4—S10	1.733 (7)	C32—S5	1.768 (7)
C4—H4A	0.9600	C32—H32A	0.9600
C4—H4B	0.9600	C32—H32B	0.9600
C4—H4C	0.9600	C32—H32C	0.9600
C5—S9	1.785 (7)	Mo1—S17	2.1156 (18)
C5—H5A	0.9600	Mo1—S35	2.1925 (16)
C5—H5B	0.9600	Mo1—S19	2.2256 (18)
C5—H5C	0.9600	Mo1—S18	2.2512 (18)
C6—S9	1.720 (7)	Mo1—Ag6^ii^	2.9915 (6)
C6—H6A	0.9600	Mo2—S36	2.1308 (19)
C6—H6B	0.9600	Mo2—S22	2.1977 (18)
C6—H6C	0.9600	Mo2—S20	2.2070 (18)
C7—S11	1.765 (7)	Mo2—S21	2.2572 (17)
C7—H7A	0.9600	Mo3—S37	2.1655 (17)
C7—H7B	0.9600	Mo3—S23	2.1855 (18)
C7—H7C	0.9600	Mo3—S25	2.2004 (18)
C8—S11	1.722 (7)	Mo3—S24	2.2480 (17)
C8—H8A	0.9600	Mo4—S38	2.1475 (18)
C8—H8B	0.9600	Mo4—S28	2.2058 (18)
C8—H8C	0.9600	Mo4—S27	2.2172 (17)
C9—S12	1.838 (8)	Mo4—S26	2.2183 (17)
C9—H9A	0.9600	Mo5—S39	2.1552 (18)
C9—H9B	0.9600	Mo5—S29	2.1988 (17)
C9—H9C	0.9600	Mo5—S31	2.2061 (18)
C10—S12	1.789 (7)	Mo5—S30	2.2693 (17)
C10—H10A	0.9600	Mo6—S40	2.1440 (17)
C10—H10B	0.9600	Mo6—S32	2.2011 (18)
C10—H10C	0.9600	Mo6—S34	2.2098 (17)
C11—S13	1.812 (7)	Mo6—S33	2.2611 (17)
C11—H11A	0.9600	O1—S1	1.536 (5)
C11—H11B	0.9600	O1—Pr1	2.411 (5)
C11—H11C	0.9600	O2—S2	1.441 (6)
C12—S13	1.611 (7)	O2—Pr1	2.423 (5)
C12—H12A	0.9600	O3—S3	1.540 (5)
C12—H12B	0.9600	O3—Pr1	2.472 (5)
C12—H12C	0.9600	O4—S4	1.558 (5)
C13—S15	1.785 (7)	O4—Pr1	2.449 (5)
C13—H13A	0.9600	O5—S5	1.495 (5)
C13—H13B	0.9600	O5—Pr1	2.513 (5)
C13—H13C	0.9600	O6—S6	1.534 (5)
C14—S15	1.689 (7)	O6—Pr1	2.486 (5)
C14—H14A	0.9600	O7—S7	1.446 (6)
C14—H14B	0.9600	O7—Pr1	2.521 (5)
C14—H14C	0.9600	O8—S8	1.580 (5)
C15—S14	1.709 (7)	O8—Pr1	2.379 (5)
C15—H15A	0.9600	O9—S9	1.527 (5)
C15—H15B	0.9600	O9—Pr2	2.462 (5)
C15—H15C	0.9600	O10—S10	1.461 (6)
C16—S14	1.755 (7)	O10—Pr2	2.436 (5)
C16—H16A	0.9600	O11—S11	1.522 (5)
C16—H16B	0.9600	O11—Pr2	2.420 (5)
C16—H16C	0.9600	O12—S12	1.506 (5)
C17—S8	1.780 (8)	O12—Pr2	2.551 (5)
C17—H17A	0.9600	O13—S13	1.512 (5)
C17—H17B	0.9600	O13—Pr2	2.458 (5)
C17—H17C	0.9600	O14—S14	1.532 (5)
C18—S8	1.932 (9)	O14—Pr2	2.399 (5)
C18—H18A	0.9600	O15—S15	1.449 (6)
C18—H18B	0.9600	O15—Pr2	2.472 (5)
C18—H18C	0.9600	O16—S16	1.476 (5)
C19—S7	1.692 (7)	O16—Pr2	2.442 (5)
C19—H19A	0.9600	S18—Ag6^ii^	2.7032 (16)
C19—H19B	0.9600	S35—Ag6^ii^	2.4929 (17)
C19—H19C	0.9600		
			
S20—Ag1—S19	120.61 (6)	S2—C29—H29C	109.5
S20—Ag1—S21	94.74 (5)	H29A—C29—H29C	109.5
S19—Ag1—S21	126.66 (5)	H29B—C29—H29C	109.5
S20—Ag1—S18	116.60 (6)	S2—C30—H30A	109.5
S19—Ag1—S18	90.53 (5)	S2—C30—H30B	109.5
S21—Ag1—S18	108.88 (5)	H30A—C30—H30B	109.5
S20—Ag1—Mo2	47.03 (4)	S2—C30—H30C	109.5
S19—Ag1—Mo2	146.88 (4)	H30A—C30—H30C	109.5
S21—Ag1—Mo2	47.79 (4)	H30B—C30—H30C	109.5
S18—Ag1—Mo2	122.58 (4)	S5—C31—H31A	109.5
S20—Ag1—Mo1	146.81 (4)	S5—C31—H31B	109.5
S19—Ag1—Mo1	46.95 (4)	H31A—C31—H31B	109.5
S21—Ag1—Mo1	117.22 (4)	S5—C31—H31C	109.5
S18—Ag1—Mo1	46.91 (4)	H31A—C31—H31C	109.5
Mo2—Ag1—Mo1	161.995 (19)	H31B—C31—H31C	109.5
S22—Ag2—S21	94.72 (5)	S5—C32—H32A	109.5
S22—Ag2—S23	128.21 (6)	S5—C32—H32B	109.5
S21—Ag2—S23	115.56 (6)	H32A—C32—H32B	109.5
S22—Ag2—S24	110.98 (6)	S5—C32—H32C	109.5
S21—Ag2—S24	115.23 (5)	H32A—C32—H32C	109.5
S23—Ag2—S24	93.50 (5)	H32B—C32—H32C	109.5
S22—Ag2—Mo2	46.82 (4)	S17—Mo1—S35	107.99 (7)
S21—Ag2—Mo2	48.21 (4)	S17—Mo1—S19	108.51 (7)
S23—Ag2—Mo2	145.24 (4)	S35—Mo1—S19	107.94 (6)
S24—Ag2—Mo2	120.96 (4)	S17—Mo1—S18	109.85 (7)
S22—Ag2—Mo3	131.56 (4)	S35—Mo1—S18	112.58 (7)
S21—Ag2—Mo3	132.93 (4)	S19—Mo1—S18	109.86 (7)
S23—Ag2—Mo3	46.23 (4)	S17—Mo1—Ag1	104.89 (5)
S24—Ag2—Mo3	47.56 (4)	S35—Mo1—Ag1	146.79 (5)
Mo2—Ag2—Mo3	168.497 (18)	S19—Mo1—Ag1	55.92 (4)
S24—Ag3—S26	141.66 (6)	S18—Mo1—Ag1	58.70 (4)
S24—Ag3—S25	93.75 (5)	S17—Mo1—Ag6^ii^	140.18 (5)
S26—Ag3—S25	108.70 (5)	S35—Mo1—Ag6^ii^	54.91 (5)
S24—Ag3—S27	92.18 (5)	S19—Mo1—Ag6^ii^	111.04 (5)
S26—Ag3—S27	89.26 (5)	S18—Mo1—Ag6^ii^	60.12 (4)
S25—Ag3—S27	141.10 (5)	Ag1—Mo1—Ag6^ii^	100.784 (18)
S24—Ag3—Mo3	47.68 (4)	S36—Mo2—S22	107.70 (7)
S26—Ag3—Mo3	142.00 (4)	S36—Mo2—S20	107.85 (7)
S25—Ag3—Mo3	46.11 (4)	S22—Mo2—S20	108.22 (7)
S27—Ag3—Mo3	128.52 (4)	S36—Mo2—S21	108.27 (7)
S24—Ag3—Mo4	132.81 (4)	S22—Mo2—S21	111.88 (7)
S26—Ag3—Mo4	46.38 (4)	S20—Mo2—S21	112.73 (6)
S25—Ag3—Mo4	131.03 (4)	S36—Mo2—Ag2	129.44 (6)
S27—Ag3—Mo4	45.02 (4)	S22—Mo2—Ag2	56.30 (5)
Mo3—Ag3—Mo4	169.415 (18)	S20—Mo2—Ag2	122.65 (5)
S29—Ag4—S28	123.51 (6)	S21—Mo2—Ag2	56.00 (4)
S29—Ag4—S30	94.87 (5)	S36—Mo2—Ag1	121.01 (6)
S28—Ag4—S30	125.49 (5)	S22—Mo2—Ag1	131.19 (5)
S29—Ag4—S27	116.20 (5)	S20—Mo2—Ag1	55.61 (5)
S28—Ag4—S27	89.13 (5)	S21—Mo2—Ag1	57.22 (4)
S30—Ag4—S27	108.32 (5)	Ag2—Mo2—Ag1	91.783 (17)
S29—Ag4—Mo5	46.80 (4)	S37—Mo3—S23	107.12 (7)
S28—Ag4—Mo5	148.13 (4)	S37—Mo3—S25	107.32 (7)
S30—Ag4—Mo5	48.10 (4)	S23—Mo3—S25	109.73 (7)
S27—Ag4—Mo5	122.72 (4)	S37—Mo3—S24	106.76 (7)
S29—Ag4—Mo4	148.46 (4)	S23—Mo3—S24	113.03 (7)
S28—Ag4—Mo4	46.64 (4)	S25—Mo3—S24	112.54 (7)
S30—Ag4—Mo4	114.79 (4)	S37—Mo3—Ag2	127.82 (5)
S27—Ag4—Mo4	46.10 (4)	S23—Mo3—Ag2	56.62 (5)
Mo5—Ag4—Mo4	159.892 (19)	S25—Mo3—Ag2	124.84 (5)
S30—Ag5—S32	115.41 (6)	S24—Mo3—Ag2	56.81 (5)
S30—Ag5—S31	95.10 (5)	S37—Mo3—Ag3	124.32 (5)
S32—Ag5—S31	127.52 (6)	S23—Mo3—Ag3	128.57 (5)
S30—Ag5—S33	115.80 (5)	S25—Mo3—Ag3	57.62 (5)
S32—Ag5—S33	94.37 (5)	S24—Mo3—Ag3	54.99 (4)
S31—Ag5—S33	110.02 (5)	Ag2—Mo3—Ag3	88.511 (17)
S30—Ag5—Mo5	48.58 (4)	S38—Mo4—S28	108.27 (7)
S32—Ag5—Mo5	144.96 (4)	S38—Mo4—S27	108.51 (7)
S31—Ag5—Mo5	46.86 (4)	S28—Mo4—S27	110.40 (7)
S33—Ag5—Mo5	120.50 (4)	S38—Mo4—S26	108.45 (7)
S30—Ag5—Mo6	133.54 (4)	S28—Mo4—S26	108.77 (7)
S32—Ag5—Mo6	46.75 (4)	S27—Mo4—S26	112.34 (7)
S31—Ag5—Mo6	130.61 (4)	S38—Mo4—Ag4	102.24 (5)
S33—Ag5—Mo6	47.88 (4)	S28—Mo4—Ag4	56.00 (4)
Mo5—Ag5—Mo6	168.289 (18)	S27—Mo4—Ag4	59.76 (5)
S35^i^—Ag6—S33	142.30 (5)	S26—Mo4—Ag4	149.03 (5)
S35^i^—Ag6—S34	106.34 (5)	S38—Mo4—Ag3	141.42 (5)
S33—Ag6—S34	94.19 (5)	S28—Mo4—Ag3	110.08 (5)
S35^i^—Ag6—S18^i^	90.61 (5)	S27—Mo4—Ag3	61.19 (5)
S33—Ag6—S18^i^	93.05 (5)	S26—Mo4—Ag3	54.42 (5)
S34—Ag6—S18^i^	140.62 (5)	Ag4—Mo4—Ag3	102.397 (17)
S35^i^—Ag6—Mo6	140.68 (4)	S39—Mo5—S29	108.20 (7)
S33—Ag6—Mo6	47.93 (4)	S39—Mo5—S31	107.24 (7)
S34—Ag6—Mo6	46.28 (4)	S29—Mo5—S31	107.97 (7)
S18^i^—Ag6—Mo6	128.66 (4)	S39—Mo5—S30	108.09 (7)
S35^i^—Ag6—Mo1^i^	46.02 (4)	S29—Mo5—S30	112.76 (6)
S33—Ag6—Mo1^i^	134.10 (4)	S31—Mo5—S30	112.37 (6)
S34—Ag6—Mo1^i^	129.93 (4)	S39—Mo5—Ag5	129.35 (6)
S18^i^—Ag6—Mo1^i^	46.23 (4)	S29—Mo5—Ag5	122.40 (5)
Mo6—Ag6—Mo1^i^	170.066 (18)	S31—Mo5—Ag5	57.17 (4)
S16—C1—H1A	109.5	S30—Mo5—Ag5	55.67 (4)
S16—C1—H1B	109.5	S39—Mo5—Ag4	122.40 (6)
H1A—C1—H1B	109.5	S29—Mo5—Ag4	55.80 (4)
S16—C1—H1C	109.5	S31—Mo5—Ag4	130.26 (5)
H1A—C1—H1C	109.5	S30—Mo5—Ag4	57.00 (4)
H1B—C1—H1C	109.5	Ag5—Mo5—Ag4	90.310 (16)
S16—C2—H2A	109.5	S40—Mo6—S32	107.85 (7)
S16—C2—H2B	109.5	S40—Mo6—S34	106.73 (6)
H2A—C2—H2B	109.5	S32—Mo6—S34	109.00 (7)
S16—C2—H2C	109.5	S40—Mo6—S33	106.70 (7)
H2A—C2—H2C	109.5	S32—Mo6—S33	112.80 (7)
H2B—C2—H2C	109.5	S34—Mo6—S33	113.42 (6)
S10—C3—H3A	109.5	S40—Mo6—Ag5	128.13 (5)
S10—C3—H3B	109.5	S32—Mo6—Ag5	56.16 (5)
H3A—C3—H3B	109.5	S34—Mo6—Ag5	125.10 (5)
S10—C3—H3C	109.5	S33—Mo6—Ag5	57.01 (4)
H3A—C3—H3C	109.5	S40—Mo6—Ag6	123.01 (5)
H3B—C3—H3C	109.5	S32—Mo6—Ag6	129.13 (5)
S10—C4—H4A	109.5	S34—Mo6—Ag6	57.99 (4)
S10—C4—H4B	109.5	S33—Mo6—Ag6	55.45 (5)
H4A—C4—H4B	109.5	Ag5—Mo6—Ag6	89.684 (16)
S10—C4—H4C	109.5	S1—O1—Pr1	133.9 (3)
H4A—C4—H4C	109.5	S2—O2—Pr1	138.2 (3)
H4B—C4—H4C	109.5	S3—O3—Pr1	127.9 (3)
S9—C5—H5A	109.5	S4—O4—Pr1	128.1 (3)
S9—C5—H5B	109.5	S5—O5—Pr1	124.1 (3)
H5A—C5—H5B	109.5	S6—O6—Pr1	126.6 (3)
S9—C5—H5C	109.5	S7—O7—Pr1	132.9 (3)
H5A—C5—H5C	109.5	S8—O8—Pr1	114.5 (3)
H5B—C5—H5C	109.5	S9—O9—Pr2	138.3 (3)
S9—C6—H6A	109.5	S10—O10—Pr2	125.7 (3)
S9—C6—H6B	109.5	S11—O11—Pr2	137.1 (3)
H6A—C6—H6B	109.5	S12—O12—Pr2	132.2 (3)
S9—C6—H6C	109.5	S13—O13—Pr2	130.7 (3)
H6A—C6—H6C	109.5	S14—O14—Pr2	130.4 (3)
H6B—C6—H6C	109.5	S15—O15—Pr2	134.5 (3)
S11—C7—H7A	109.5	S16—O16—Pr2	134.3 (3)
S11—C7—H7B	109.5	O8—Pr1—O1	81.92 (17)
H7A—C7—H7B	109.5	O8—Pr1—O2	72.56 (19)
S11—C7—H7C	109.5	O1—Pr1—O2	78.30 (16)
H7A—C7—H7C	109.5	O8—Pr1—O4	121.32 (17)
H7B—C7—H7C	109.5	O1—Pr1—O4	142.40 (17)
S11—C8—H8A	109.5	O2—Pr1—O4	81.43 (18)
S11—C8—H8B	109.5	O8—Pr1—O3	146.88 (17)
H8A—C8—H8B	109.5	O1—Pr1—O3	74.52 (17)
S11—C8—H8C	109.5	O2—Pr1—O3	80.02 (17)
H8A—C8—H8C	109.5	O4—Pr1—O3	70.98 (16)
H8B—C8—H8C	109.5	O8—Pr1—O6	71.49 (18)
S12—C9—H9A	109.5	O1—Pr1—O6	147.39 (17)
S12—C9—H9B	109.5	O2—Pr1—O6	110.03 (17)
H9A—C9—H9B	109.5	O4—Pr1—O6	69.76 (16)
S12—C9—H9C	109.5	O3—Pr1—O6	137.17 (16)
H9A—C9—H9C	109.5	O8—Pr1—O5	138.89 (18)
H9B—C9—H9C	109.5	O1—Pr1—O5	110.22 (17)
S12—C10—H10A	109.5	O2—Pr1—O5	147.11 (18)
S12—C10—H10B	109.5	O4—Pr1—O5	72.98 (18)
H10A—C10—H10B	109.5	O3—Pr1—O5	72.42 (17)
S12—C10—H10C	109.5	O6—Pr1—O5	80.34 (17)
H10A—C10—H10C	109.5	O8—Pr1—O7	72.15 (18)
H10B—C10—H10C	109.5	O1—Pr1—O7	76.09 (15)
S13—C11—H11A	109.5	O2—Pr1—O7	138.67 (18)
S13—C11—H11B	109.5	O4—Pr1—O7	136.26 (17)
H11A—C11—H11B	109.5	O3—Pr1—O7	122.48 (15)
S13—C11—H11C	109.5	O6—Pr1—O7	77.98 (14)
H11A—C11—H11C	109.5	O5—Pr1—O7	73.15 (18)
H11B—C11—H11C	109.5	O14—Pr2—O11	146.54 (17)
S13—C12—H12A	109.5	O14—Pr2—O10	120.58 (17)
S13—C12—H12B	109.5	O11—Pr2—O10	78.64 (17)
H12A—C12—H12B	109.5	O14—Pr2—O16	70.99 (17)
S13—C12—H12C	109.5	O11—Pr2—O16	142.44 (17)
H12A—C12—H12C	109.5	O10—Pr2—O16	78.48 (19)
H12B—C12—H12C	109.5	O14—Pr2—O13	73.84 (17)
S15—C13—H13A	109.5	O11—Pr2—O13	73.25 (17)
S15—C13—H13B	109.5	O10—Pr2—O13	131.73 (18)
H13A—C13—H13B	109.5	O16—Pr2—O13	142.69 (17)
S15—C13—H13C	109.5	O14—Pr2—O9	136.28 (18)
H13A—C13—H13C	109.5	O11—Pr2—O9	73.54 (19)
H13B—C13—H13C	109.5	O10—Pr2—O9	71.19 (17)
S15—C14—H14A	109.5	O16—Pr2—O9	71.07 (18)
S15—C14—H14B	109.5	O13—Pr2—O9	132.93 (17)
H14A—C14—H14B	109.5	O14—Pr2—O15	73.77 (16)
S15—C14—H14C	109.5	O11—Pr2—O15	102.65 (17)
H14A—C14—H14C	109.5	O10—Pr2—O15	151.40 (18)
H14B—C14—H14C	109.5	O16—Pr2—O15	84.38 (18)
S14—C15—H15A	109.5	O13—Pr2—O15	74.16 (18)
S14—C15—H15B	109.5	O9—Pr2—O15	81.69 (18)
H15A—C15—H15B	109.5	O14—Pr2—O12	78.50 (16)
S14—C15—H15C	109.5	O11—Pr2—O12	86.49 (17)
H15A—C15—H15C	109.5	O10—Pr2—O12	66.42 (17)
H15B—C15—H15C	109.5	O16—Pr2—O12	110.71 (17)
S14—C16—H16A	109.5	O13—Pr2—O12	73.36 (17)
S14—C16—H16B	109.5	O9—Pr2—O12	135.87 (17)
H16A—C16—H16B	109.5	O15—Pr2—O12	141.90 (17)
S14—C16—H16C	109.5	O1—S1—C21	109.4 (2)
H16A—C16—H16C	109.5	O1—S1—C22	104.3 (2)
H16B—C16—H16C	109.5	C21—S1—C22	100.7 (3)
S8—C17—H17A	109.5	O2—S2—C30	103.6 (3)
S8—C17—H17B	109.5	O2—S2—C29	110.9 (3)
H17A—C17—H17B	109.5	C30—S2—C29	95.5 (3)
S8—C17—H17C	109.5	O3—S3—C23	101.1 (3)
H17A—C17—H17C	109.5	O3—S3—C24	99.1 (3)
H17B—C17—H17C	109.5	C23—S3—C24	101.0 (4)
S8—C18—H18A	109.5	O4—S4—C25	106.0 (2)
S8—C18—H18B	109.5	O4—S4—C26	103.2 (3)
H18A—C18—H18B	109.5	C25—S4—C26	97.7 (4)
S8—C18—H18C	109.5	O5—S5—C32	105.9 (3)
H18A—C18—H18C	109.5	O5—S5—C31	101.7 (2)
H18B—C18—H18C	109.5	C32—S5—C31	94.2 (4)
S7—C19—H19A	109.5	O6—S6—C28	104.2 (3)
S7—C19—H19B	109.5	O6—S6—C27	101.7 (2)
H19A—C19—H19B	109.5	C28—S6—C27	100.2 (4)
S7—C19—H19C	109.5	O7—S7—C20	105.3 (3)
H19A—C19—H19C	109.5	O7—S7—C19	107.1 (4)
H19B—C19—H19C	109.5	C20—S7—C19	98.4 (5)
S7—C20—H20A	109.5	O8—S8—C17	104.8 (2)
S7—C20—H20B	109.5	O8—S8—C18	103.2 (3)
H20A—C20—H20B	109.5	C17—S8—C18	94.2 (4)
S7—C20—H20C	109.5	O9—S9—C6	105.0 (2)
H20A—C20—H20C	109.5	O9—S9—C5	101.8 (3)
H20B—C20—H20C	109.5	C6—S9—C5	96.3 (4)
S1—C21—H21A	109.5	O10—S10—C4	101.4 (3)
S1—C21—H21B	109.5	O10—S10—C3	105.3 (3)
H21A—C21—H21B	109.5	C4—S10—C3	99.8 (4)
S1—C21—H21C	109.5	O11—S11—C8	104.2 (3)
H21A—C21—H21C	109.5	O11—S11—C7	105.8 (3)
H21B—C21—H21C	109.5	C8—S11—C7	93.7 (3)
S1—C22—H22A	109.5	O12—S12—C10	106.2 (3)
S1—C22—H22B	109.5	O12—S12—C9	97.6 (3)
H22A—C22—H22B	109.5	C10—S12—C9	95.6 (4)
S1—C22—H22C	109.5	O13—S13—C12	108.6 (3)
H22A—C22—H22C	109.5	O13—S13—C11	105.7 (3)
H22B—C22—H22C	109.5	C12—S13—C11	101.8 (4)
S3—C23—H23A	109.5	O14—S14—C15	106.1 (3)
S3—C23—H23B	109.5	O14—S14—C16	107.3 (3)
H23A—C23—H23B	109.5	C15—S14—C16	97.8 (4)
S3—C23—H23C	109.5	O15—S15—C14	107.7 (3)
H23A—C23—H23C	109.5	O15—S15—C13	99.9 (3)
H23B—C23—H23C	109.5	C14—S15—C13	99.0 (3)
S3—C24—H24A	109.5	O16—S16—C1	103.9 (2)
S3—C24—H24B	109.5	O16—S16—C2	106.1 (2)
H24A—C24—H24B	109.5	C1—S16—C2	98.6 (4)
S3—C24—H24C	109.5	Mo1—S18—Ag1	74.39 (5)
H24A—C24—H24C	109.5	Mo1—S18—Ag6^ii^	73.65 (5)
H24B—C24—H24C	109.5	Ag1—S18—Ag6^ii^	118.71 (6)
S4—C25—H25A	109.5	Mo1—S19—Ag1	77.13 (5)
S4—C25—H25B	109.5	Mo2—S20—Ag1	77.36 (5)
H25A—C25—H25B	109.5	Mo2—S21—Ag2	75.79 (5)
S4—C25—H25C	109.5	Mo2—S21—Ag1	74.99 (5)
H25A—C25—H25C	109.5	Ag2—S21—Ag1	112.64 (6)
H25B—C25—H25C	109.5	Mo2—S22—Ag2	76.88 (5)
S4—C26—H26A	109.5	Mo3—S23—Ag2	77.15 (5)
S4—C26—H26B	109.5	Mo3—S24—Ag3	77.32 (5)
H26A—C26—H26B	109.5	Mo3—S24—Ag2	75.63 (5)
S4—C26—H26C	109.5	Ag3—S24—Ag2	110.04 (6)
H26A—C26—H26C	109.5	Mo3—S25—Ag3	76.27 (5)
H26B—C26—H26C	109.5	Mo4—S26—Ag3	79.20 (5)
S6—C27—H27A	109.5	Mo4—S27—Ag4	74.15 (5)
S6—C27—H27B	109.5	Mo4—S27—Ag3	73.79 (5)
H27A—C27—H27B	109.5	Ag4—S27—Ag3	118.81 (6)
S6—C27—H27C	109.5	Mo4—S28—Ag4	77.36 (5)
H27A—C27—H27C	109.5	Mo5—S29—Ag4	77.40 (5)
H27B—C27—H27C	109.5	Mo5—S30—Ag5	75.75 (5)
S6—C28—H28A	109.5	Mo5—S30—Ag4	74.90 (5)
S6—C28—H28B	109.5	Ag5—S30—Ag4	110.99 (6)
H28A—C28—H28B	109.5	Mo5—S31—Ag5	75.98 (5)
S6—C28—H28C	109.5	Mo6—S32—Ag5	77.10 (5)
H28A—C28—H28C	109.5	Mo6—S33—Ag6	76.62 (5)
H28B—C28—H28C	109.5	Mo6—S33—Ag5	75.10 (5)
S2—C29—H29A	109.5	Ag6—S33—Ag5	110.69 (6)
S2—C29—H29B	109.5	Mo6—S34—Ag6	75.73 (5)
H29A—C29—H29B	109.5	Mo1—S35—Ag6^ii^	79.07 (5)

Symmetry codes: (i) *x*, *y*, *z*+1; (ii) *x*, *y*, *z*−1.
